# True open access to urological science at the International Brazilian Journal of Urology

**DOI:** 10.1590/S1677-5538.IBJU.2023.04.02

**Published:** 2023-08-07

**Affiliations:** 1 Universidade de São Paulo - USP Departamento de Urologia São Paulo SP Brasil Departamento de Urologia, Universidade de São Paulo - USP, São Paulo, SP, Brasil; 2 Universidade Estadual de Campinas Campinas SP Brasil UroScience, Universidade Estadual de Campinas, Unicamp, Campinas, SP, Brasil

The recent resignation of the entire editorial board of Neuroimage (IF 7.4) - one of the most prominent scientific journals dedicated to brain imaging research - rekindles the discussion on access to scientific data and the high costs for publication and access to scientific data (1).

The episode highlights the disproportion between the high costs that researchers must pay to publish their research and the exorbitant profit margins of large publishers. It is worth remembering two very important aspects: (1) The entire peer review process that guarantees the quality of publications is carried out by the most distinguished researchers, who receive nothing for this difficult and demanding work; (2) Most of the scientific work produced by academic researchers is funded by government institutions and often part of the costs come out of the researchers’ pockets.

Faced with these facts, the mass resignation of more than 40 renowned scientists from the editorial board of a renowned scientific journal was applauded by a large part of the scientific community. The resignation came in protest at what former publishers describe as the “greed” of a publishing giant after the company refused to cut publishing costs despite the company’s profit margins that are estimated to be close to 40%, according to 2019 data. Former editors say this is “unethical” and unrelated to the costs involved.

At the International Brazilian Journal of Urology (IBJU), we are proud to offer true open access to Science, based on principles and practices that assure that the most relevant research in urology is widely distributed online, free of access charges or other barriers, like green open access.

The IBJU is peer-reviewed and have a rigorous editorial process to maintain quality and ensure accuracy. It is partially supported by the Brazilian Ministry of Science and Technology, National Council for Scientific and Technological Development (CNPQ) and Coordination for the Improvement of Higher Education Personnel (CAPES), which makes possible not charging authors an article processing fee (APC) to cover publication costs. The mean cost for every published article is US$30,00 dollars.

To the best of our knowledge the IBJU is the unicorn of open access among Urological journals, as PubMed indexed, highest official impact factor (IF 3.05), and no APC cost for authors.

## References

[1] (2023). Journal The Guardian. Too greedy’: mass walkout at global science journal over ‘unethical’ fees.

